# DOES INCREASING LIGHT PHYSICAL ACTIVITY IN MIDLIFE AND OLD AGE BUFFER AGAINST COGNITIVE DECLINES?

**DOI:** 10.1093/geroni/igad104.0363

**Published:** 2023-12-21

**Authors:** Jeremy Hamm, Kelly Parker, Margie Lachman, Jacqueline Mogle, Katherine Duggan, Ryan McGrath

**Affiliations:** North Dakota State University, Fargo, North Dakota, United States; North Dakota State University, Fargo, North Dakota, United States; Brandeis University, Waltham, Massachusetts, United States; Clemson University, Clemson, South Carolina, United States; North Dakota State University, Fargo, North Dakota, United States; North Dakota State University, Fargo, North Dakota, United States

## Abstract

Although it is well established that moderate-to-vigorous physical activity (MVPA) buffers against cognitive declines, less is known about the benefits of lower intensity physical activities that are more feasible, inclusive, and amenable to change in old age. Our study examined the extent to which increases in light physical activity protected against longitudinal declines in cognitive functioning and whether such a relationship become pronounced in old age when opportunities for MVPA are often reduced. We analyzed 9-year data from the national Midlife in the United States Study (n=2,229; age=56±11.13 years) using regression models that assessed whether change in light physical activity predicted corresponding changes in episodic memory and executive functioning for adults across the lifespan. All models controlled for age, sex, socioeconomic status, functional status, and moderate and vigorous physical activity. Beyond more vigorous forms of physical activity, increasing light physical activity predicted less decline in episodic memory (β=.06, p=.010) and executive functioning (β=.15, p<.001) over the 9-year follow-up period. Effect sizes for moderate and vigorous physical activity on episodic memory (βs=.01-.02) and executive functioning (βs=.03) were less than half that observed for light physical activity. Moderation models showed that, for episodic memory, the benefits of increasing light physical activity were pronounced at older ages. Findings provide initial evidence that increasing light physical activity may help slow age-related declines in episodic memory and executive functioning and that its protective influence may become paramount in later life when individuals commonly encounter constraints that can limit their capacity to engage in MVPA.

